# Simulation model to assess the validity of the clinical portfolio diet score used in the PortfolioDiet.app for dietary self-tracking: a secondary analysis of a randomized controlled trial in hyperlipidemic adults

**DOI:** 10.3389/fnut.2024.1398450

**Published:** 2024-08-07

**Authors:** Meaghan E. Kavanagh, Andrea J. Glenn, Laura Chiavaroli, Gloria A. Morgan, Robert G. Josse, Vasanti S. Malik, Christopher P. F. Marinangeli, Cyril W. C. Kendall, David J. A. Jenkins, John L. Sievenpiper

**Affiliations:** ^1^Department of Nutritional Sciences, Temerty Faculty of Medicine, University of Toronto, Toronto, ON, Canada; ^2^Toronto 3D Knowledge Synthesis and Clinical Trials Unit, Clinical Nutrition and Risk Factor Modification Center, St. Michael’s Hospital, Toronto, ON, Canada; ^3^Department of Nutrition, Harvard T.H. Chan School of Public Health, Boston, MA, United States; ^4^Li Ka Shing Knowledge Institute, St. Michael’s Hospital, Toronto, ON, Canada; ^5^Division of Endocrinology and Metabolism, St. Michael’s Hospital, Toronto, ON, Canada; ^6^Department of Medicine, Temerty Faculty of Medicine, University of Toronto, Toronto, ON, Canada; ^7^Protein Industries Canada, Regina, SK, Canada; ^8^College of Pharmacy and Nutrition, University of Saskatchewan, Saskatoon, SK, Canada

**Keywords:** nutrition, portfolio diet, dietary patterns, lipids, cholesterol reduction, self-monitoring

## Abstract

**Introduction:**

The Portfolio Diet combines cholesterol-lowering plant foods for the management of cardiovascular disease risk. However, the translation of this dietary approach into clinical practice necessitates a user-friendly method for patients to autonomously monitor their adherence.

**Objective:**

This study aimed to develop and validate the clinical-Portfolio Diet Score (c-PDS) as a food-based metric to facilitate self-tracking of the Portfolio Diet.

**Methods:**

Using a simulation model to estimate the c-PDS, the validity was assessed in a secondary analysis of a completed trial of the Portfolio Diet in 98 participants with hyperlipidemia over 6 months. Concurrent and predictive validity of the estimated c-PDS were assessed against the reference measure (weighed 7-day diet records) and concomitant changes in LDL-C from baseline to 6 months. Bland–Altman analysis was used to assess the limits of agreement between the two methods.

**Results:**

The c-PDS was positively correlated with dietary adherence as measured using the 7-day diet records (*r* = 0.94, *p* < 0.001). The c-PDS was negatively correlated with change in LDL-C (*r* = −0.43, *p* < 0.001) with a 1-point increase in the c-PDS being associated with a − 0.04 mmol/L (CI:−0.06,−0.03; *p* < 0.001) or a 1.09% reduction in LDL-C. Visual evaluation of the Bland–Altman plots showed reasonable agreement.

**Conclusion:**

These findings indicate good validity of the c-PDS for primary prevention in adults with hyperlipidemia. The predictive validity findings have informed the goals and messaging within the PortfolioDiet.app, a digital health application for delivering the Portfolio Diet. Future research will assess the effectiveness of the intended combination of the c-PDS and the PortfolioDiet.app in supporting behavior change.

## 1 Introduction

Cardiovascular diseases (CVD) are the leading cause of death globally (1). Despite notable advancements in the medical field, CVD continues to escalate, underscoring the need for innovative primary and secondary prevention strategies. Dietary modification has been consistently established as front-line therapy for management of CVD risk factors such as dyslipidemia (2) and as a leading strategy for population prevention of CVD (3, 4). The Portfolio Diet is a nutrition therapy which has high quality evidence for lowering LDL-C in adults with hyperlipidemia (5, 6). Initially developed as a “portfolio” of foods with established cholesterol-lowering properties, the diet combines nuts and seeds, viscous fiber sources, plant protein sources (soy and dietary pulses), plant sterols, and plant-derived monounsaturated fatty acids (MUFA) sources. Despite the demonstrated efficacy of the Portfolio Diet on established cardiovascular parameters (7) and its recognition in clinical practice guidelines (8–12), the Portfolio Diet’s integration into clinical practice faces several barriers, a challenge commonly shared with other dietary approaches.

There are many aspects of a nutrition trial environment that are not easily replicated in clinical settings therefore tools are needed to translate nutrition interventions from trials to a “real-world” environment. Simple scoring methods can facilitate self-monitoring and be used to provide adherence-related feedback to participants (13). However, no scoring system or self-tracking tool is available for the Portfolio Diet. To help translate current clinical practice guidelines for dyslipidemia and enable the Portfolio Diet to be of use in clinical settings, a tested user-friendly score was needed. The objective of this research was to validate the clinical-Portfolio Diet Score (c-PDS) as a food-based metric to facilitate (self-) tracking of the Portfolio Diet by participants and clinicians. The score’s concurrent validity for measuring adherence to the Portfolio Diet was assessed against an established reference measure of 7-day diet records (7DDR) and the score’s predictive validity was assessed with a biomarker of adherence to the diet, LDL-C.

## 2 Methods

### 2.1 Study design

The validity of the c-PDS was assessed within a completed 6-month randomized controlled trial of the Portfolio Diet in primary prevention hyperlipidemic adults (ClinicalTrials.gov identifier, NCT00438425). The trial compared a low-saturated fat therapeutic diet (control) to the Portfolio Diet, where counseling was delivered at two different frequencies (routine or intense). The routine Portfolio Diet intervention involved 2 clinic visits, and the intense Portfolio Diet intervention involved 7 clinic visits over the six-month intervention. Further details on the trial have been described elsewhere (6). A total of 108 participants completed the trial at the Toronto site, which is considered an acceptable sample size for validation studies (14). Of the 108 participants that completed the study, 8 had missing diet records and 2 had missing blood lipid data. Supplementary Figure S1 presents the flow of participants for analysis with a total of 98 participants included.

### 2.2 Dietary assessment

Weighed 7-day diet records (7DDR) were collected at baseline and 6 months and analyzed by ESHA Food Processor SQL (version 10.1.1; ESHA, Salem, Oregon). The reference measure was calculated using participant’s percent adherence to their prescribed Portfolio Diet based on reported total energy intake (45-g/day of nuts, 50-g/day of protein from plants [soy, pulses], 20-g/day of viscous fiber, 2-g/day of plant sterols, and 45-g/day of MUFA) based on a 2000-kcal diet. This reference method was the original way adherence to the Portfolio Diet has been assessed since 2003 (5, 6, 15), with the only difference being the inclusion of 45-g/day of MUFA as a food category which was added to the Portfolio Diet in a subsequent trial (16).

### 2.3 Development of the clinical portfolio diet score (c-PDS)

#### 2.3.1 Food-based scores

The Portfolio Diet targets were initially conceptualized as grams per day with adherence being measured using weighed 7DDRs. However, communication of nutrient targets to patients can be challenging; leading dietitians to provide food-based, instead of nutrient-based targets, when counselling patients (17). Beyond this point, weighed 7-day diet records are time consuming, and can be challenging to interpret requiring access to a detailed nutrient database and are unable to provide immediate feedback to participants if using grams per day as targets. These limitations are also true of other dietary assessment methods such as food frequency questionnaires (FFQ) or 24-h recalls. These concerns align with a Scientific Statement from the American Heart Association which called for more validated rapid diet screener tools to assess diet quality at point of care for management of CVD, with a specific focus on screeners for dietary patterns (18). To further emphasize this food-based counseling and align with current recommendations, a food-based screener was developed for use in the ongoing Portfolio Diet trial (ClinicalTrials.gov Identifier: NCT02481466). Supplementary Figure S2 provides the 2,000 kcal version of the original food-based screener. This initial version of the screener was completed by the participant, and then used by the study dietitians to provide rapid and personalized feedback. This initial screener counted traditional food servings in household measures of each Portfolio Diet food and did not provide an overall target. This screener was used as a starting point to inform the development of the c-PDS.

#### 2.3.2 The c-PDS food categories

A total of 5 Portfolio Diet categories were chosen: Nuts and Seeds, Plant Protein, Viscous Fiber, Plant Sterols, and High MUFA Oils & Foods. Target amounts for 1-point of the c-PDS were food-based and provided in reasonable household measurements. For each category of the c-PDS a maximum of 5-points was set (total score ranged from 0 to 25-points).

#### 2.3.3 Accounting for different energy needs

In previous trials, adherence to the Portfolio Diet was measured using total energy intake calculated from the weighed 7DDRs (6). As having patients track calories would complicate self-tracking, thus hindering the score’s usability, we instead used estimated energy requirement (EER) based-on the Institute of Medicine’s equations. The c-PDS targets were adjusted based on the EER. The EER accounts for four variables: sex, baseline weight, age, and physical activity level (sedentary, lightly active, moderately active, very active), and was based on the Institute of Medicine equations (19).

### 2.4 Estimating the c-PDS

To emulate participant input of dietary items, a simulation model was executed utilizing the weighed 7DDRs and participant-specific data. The c-PDS was derived by using the weighed 7DDRs while accounting for underlying assumptions of the model. Supplementary Figure S3 provides an illustration of the simulation model’s decision tree and the binary questions. The decision tree included sequences of 1 to 5 binary questions for each line of the 7DDRs. These are summarized in the list below.

1. Food item? From the 7DDRs, relevant food items were identified from non-food items. Recorded food intake from the 7DDRs was converted to household measurements (20) to reflect a diet checklist.
2. Portfolio Food? Then possible portfolio foods from non-portfolio foods were identified.
3. EER? Subsequently, using participant-specific data individual participants were allocated to respective calorie groups (1,200, 1,600, or 2000 kcal) based on their estimated EER, corresponding to adjustments of their respective c-PDS targets.
4. Category? Then the identified Portfolio Diet foods were classified into the five distinct categories: Nuts and Seeds, Plant Protein, Viscous Fiber, Plant Sterols, and High MUFA Oils and Foods.
5. Point value? Point value assigned based on household measure. Emulating user input, points values were rounded up to the nearest 0.5 with a maximum point value of 5 points per category per day, resulting in a total score ranging from 0 to 25-points.

Underlying assumptions included that the EER calculation provided a reasonable prediction of different energy needs (discussed in section 2.3.3 Accounting for different energy needs) and that the information included in the 7DDR is reflective of actual dietary intake.

This estimated c-PDS was then assessed for its concurrent and predictive validity as discussed below in the statistical analysis section.

### 2.5 LDL-C assessment

All fasting blood samples were analyzed in the routine hospital laboratory using Beckman SYNCHRON LX Systems (Mississauga, Ontario, Canada). LDL-C level was calculated using the Friedewald equation (21).

### 2.6 Statistical analysis

The control and two treatment arms were pooled together. Concurrent validity was assessed by Pearson correlation coefficient of the c-PDS (ranging from 0 to a total of 25-points), with the overall adherence to the Portfolio Diet (by percent) using weighed 7DDRs. Bland–Altman analysis was used to assess the limits of agreement between the two methods (22). Bland–Altman analysis is commonly used to verify the accuracy of a new dietary assessment measure against another established measure (23, 24). Absolute agreement is calculated by taking the mean of the differences for the two methods against the mean intake of the two methods. The 95% limits of agreement provide an interval within which 95% of these differences are expected to fall. Limits of agreement between 50 and 200% was considered reasonable (25). Bland–Altman plots were used to visualize the agreement between the two methods (26, 27).

For predictive validity, Pearson correlation coefficients were used to assess the correlation of change (from baseline to 6 month) in the c-PDS with change in LDL-C. Assumptions of normality were met for LDL-C, and the linear relationship between c-PDS and LDL-C was assessed with a scatterplot. Multiple linear regression was also used to assess the association of change in the c-PDS with concomitant changes in LDL-C after adjustment for pre-specified covariates: age (continuous), sex (male, female), ethnicity (Asian, Black, Caucasian, Hispanic, Other), body mass index (continuous), and baseline LDL-C (continuous). Effect size was calculated using β-coefficients to estimate the change in LDL-C level per 1-point and 12-point increases in the c-PDS. All statistical tests were two-tailed, and *p* < 0.05 was considered significant. Statistical analysis was performed using Stata version 17 (Stata Statistical Software: Release 17. College Station, TX, USA).

## 3 Results

### 3.1 The clinical portfolio diet score (c-PDS)

Supplementary Table S1 reports the c-PDS with the category targets in household measures for EER groups. The five categories of the Portfolio Diet each allow between 0 and 5 points based on pre-defined targets with points rounded to the nearest 0.5 resulting in a total range of 0 to 25 points (i.e., ½ cup chickpeas has ~10 grams protein = 1 point for plant protein for those with an EER of 2000 kcal). Supplementary Figure S4 provides an example of a c-PDS tracking sheet for a EER of 2000 kcal.

### 3.2 Participant characteristics

As shown in Supplementary Table S2, participants included were an average of 56 ± 9 years of age (mean ± standard deviation), predominantly female (60%), with the majority of participants identifying as White (65%) or Asian (18%). Prior to being randomized, 14% participants were on lipid-lowering medications. These medications were stopped 2 weeks before randomization. At baseline participants had a BMI of 27.1 ± 4.2 kg/m^2^, total cholesterol of 6.30 ± 0.93 mmol/L, triglycerides of 1.49 ± 0.90, HDL-C of 1.32 ± 0.34, LDL-C of 4.33 ± 0.80 mmol/L, non-HDL-C of 4.98 ± 0.89 mmol/L, and an Apolipoprotein B (ApoB) of 1.20 ± 0.19 g/L. For baseline diet participants had an average c-PDS of 3.11 ± 2.64 points.

### 3.3 Validation of the c-PDS

#### 3.3.1 Concurrent validity

**Figure 1:**
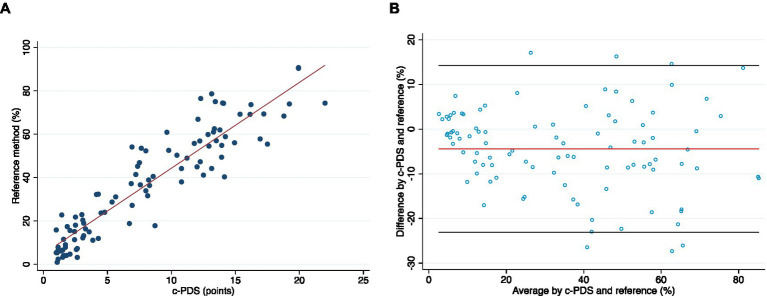
**(A)** A scatter plot of the correlation between the dietary adherence to Portfolio Diet at week 24 measured by the reference method, shown as percentage, and the clinical-Portfolio Diet Score (c-PDS), shown as points (range, 0 to 25-points). **(B)** Bland–Altman plot visually presents the agreement between the c-PDS and the reference method of % adherence to the Portfolio Diet assessed by weighed 7-day diet records (week 24). The x-axis is the mean of the two methods, and the y-axis is the difference between the two methods. The red line is the mean difference and black lines represent upper and lower 95% limits of agreement. The Bland–Altman plot demonstrates reasonable agreement between the c-PDS and the reference method by dietary records. Clustering of datapoints closest to the origin of X-axis was attributed to the control group’s low adherence to the Portfolio Diet. c-PDS, clinical-Portfolio Diet Score; LDL-C low-density lipoprotein cholesterol.

Table 1 shows the concurrent validity results with correlation coefficients for individual categories and total Portfolio Diet adherence. The c-PDS was positively correlated with adherence using the 7DDRs. Figure 1A presents a scatterplot illustrating average dietary adherence measured by the reference method, shown as percentage, and the c-PDS, shown as points. Overall, the c-PDS was positively correlated with the reference method from the 7DDRs (*r* = 0.95, *p* < 0.001). The Bland–Altman analysis demonstrated reasonable agreement with a mean difference in % dietary adherence between the c-PDS to the reference of −4.44 [95% confidence intervals (CI): −6.32, −2.55] and narrow limits of agreement (−23.10 to 14.22). Supplementary Table S3 shows results for each food category separately. Figure 1B presents the Bland–Altman plot and visually demonstrates reasonable agreement between the c-PDS and the reference method by dietary records. Clustering of datapoints closest to the origin of X-axis was attributed to the control group’s low adherence to the Portfolio Diet.

**Table 1:** Comparison of the mean intake of the Portfolio Diet measured by reference method and the clinical-Portfolio Diet Score (c-PDS) by category assessed at 6 months (*n* = 98).

Portfolio diet categories	Reference method* (95% CI)	c-PDS, points (95% CI)	Pearson Correlation Coefficient	*p*-Value
Nuts and seeds	9.49% (7.89, 11.08)	2.08 (1.72, 2.45)	0.95	<0.001
Plant protein	6.63% (5.43, 7.83)	1.51 (1.23, 1.78)	0.86	<0.001
Viscous fiber	6.54% (5.12, 7.97)	1.89 (1.56, 2.22)	0.89	<0.001
Plant sterols	6.47% (5.02, 7.92)	1.43 (1.10, 1.76)	0.97	<0.001
High MUFA oils and foods	7.71% (6.43, 8.98)	1.20 (1.03, 1.36)	0.75	<0.001
Total	36.85% (31.94, 41.76)	8.10 (6.96, 9.25)	0.92	<0.001

*Reference measure expressed as percentage of mean daily calories for each Portfolio Diet categories. c-PDS, clinical-Portfolio Diet Score; CI, confidence intervals; MUFA, monounsaturated fatty acids.

Supplementary Figures S4–S8 present side-by-side comparisons of scatter plots and Bland–Altman plots for each of the Portfolio Diet categories, with the corresponding correlation coefficients being found in Table 1. Agreement between the two methods was reasonable with category-specific Bland–Altman plots suggesting modest underestimation of reported intakes by the c-PDS for all categories except for the Viscous Fiber, where there was a modest overestimation by the c-PDS.

#### 3.3.2 Predictive validity

**Figure 2:**
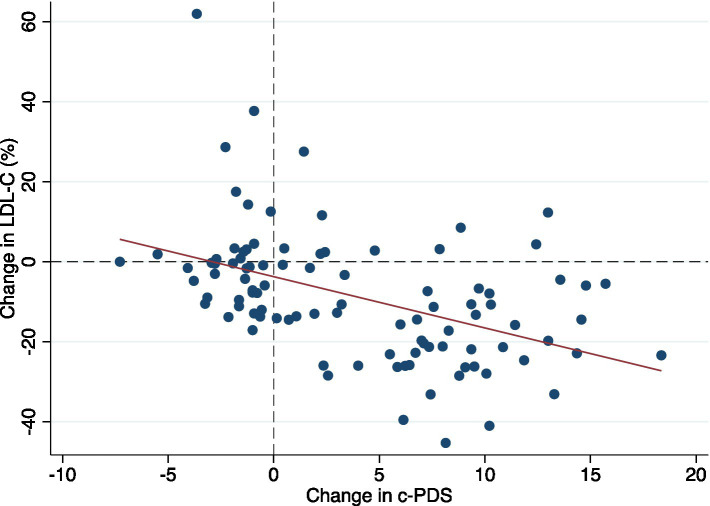
A scatter plot showing the predictive validity of the clinical-Portfolio Diet Score using % change of LDL-C over 6 months as a biomarker of adherence. c-PDS, clinical-Portfolio Diet Score; LDL-C low-density lipoprotein cholesterol.

Figure 2 presents a scatter plot of change in c-PDS and change in LDL-C (%) from baseline to 6 months. At 6 months the average LDL-C was 3.88 ± 0.65 mmol/L and c-PDS was 8.10 ± 5.7 points. The average change in the c-PDS from baseline to 6 months was 3.91 points [95% confidence intervals (CI): 2.74, 5.07] and the change in LDL-C was −0.45 mmol/L (CI: −0.55, −0.35) or − 8.75%.

Table 2 shows the linear regression for change in c-PDS and concomitant changes in LDL-C from baseline to 6 months. The c-PDS was inversely correlated with LDL-C (*r* = −0.43, *p* < 0.0001) with an apparent inverse association in the crude analysis (β coefficient: −0.06 mmol/L) (CI: −0.08, −0.03; *p* < 0.001) or a 1.28% reduction in LDL-C. In the multiple linear regression analysis, the association remained statistically significant and the c-PDS was inversely associated with concomitant change in LDL-C with a 1-point increase in score associated with a − 0.04 mmol/L (CI: −0.06, −0.03; *p* < 0.001) or a 1.09% reduction in LDL-C, after adjusting for covariates. Multiplying this estimate, a 12-point increase in the c-PDS corresponds to a reduction in LDL-C of 0.53 mmol/L (13.1%).

**Table 2:** Linear regression for the change in the clinical-Portfolio Diet Score (c-PDS) and concomitant change in LDL-C as a biomarker of adherence, over 6 months (*n* = 98).

	LDL-C (mmol/L)^a^ (95% CI)	LDL-C (%)^a^ (95% CI)	*p-*value	*R* ^2^
Unadjusted	−0.06 (−0.08, −0.03)	−1.28 (−1.78,-0.78)	<0.0001	0.21
Adjusted^b^	−0.04 (−0.06, −0.03)	−1.09 (−1.55, −0.63)	<0.0001	0.46

^a^Per 1-point increase in c-PDS. ^b^Model adjusted for age, sex, BMI, ethnicity, and baseline LDL-C. PDS based on specific foods listed in Supplementary Table S1. *R*^2^, multidimensional determination coefficient; β, coefficients.

Supplementary Table S4 shows the average change from baseline to 6 months by category and the association with change in LDL-C. Out of the five categories, Plant Protein presented the strongest inverse association, with a 1-point increase being associated with a − 0.15 mmol/L (CI: −0.28, −0.07; *p* < 0.001) or − 3.80% in LDL-C. There was no change in High MUFA Oils and Foods, and this category was not associated with change in LDL-C.

## 4 Discussion

Our secondary analysis of a completed trial in 98 primary prevention participants demonstrates that compared to reference method, the estimated c-PDS showed reasonable concurrent validity for measuring adherence to the Portfolio Diet. The mean agreement between the c-PDS and the reference method was reasonable with the limits of agreement falling within the acceptable boundaries (50–200%). As hypothesized, the change in c-PDS was negatively correlated with change in LDL-C from baseline to 6 months. A 1-point increase in the c-PDS was associated with an LDL-C reduction of 1.09% (0.04 mmol/L) after adjusting for covariates. These results overall indicate good validity of the c-PDS in adults with hyperlipidemia and allow us to estimate that a clinically meaningful reduction in LDL-C of 0.53 mmol/L (13.1%) may be observed with an achievable 12-point increase in the c-PDS.

### 4.1 Comparison with previous work

When looking at the current literature, existing tools for other dietary patterns beyond the Portfolio Diet have been developed and validated. Dietary scores and their respective short screeners have been developed and tested for rapid estimation of adherence in time-limited settings for dietary patterns such as the Mediterranean Diet (28), the Dietary Approach to Stop Hypertension (DASH) diet (29), Alternative Healthy Eating Index (AHEI) (30), and Dietary Guidelines, such as Canada’s Food Guide (31). These short screeners permit these dietary patterns to be easily assessed and used in many settings, leading to greater dissemination. As part of an dietary pattern intervention, a scoring method was used in the DASH Cloud intervention (13). In this trial a previously developed index (32) was used to facilitate self-monitoring and to communicate adherence-related feedback to participants through automated text-messages where adherence to the diet was found to increase during the trial.

Although redundancy was an important consideration when developing the c-PDS, a separate scoring system was needed for the Portfolio Diet as previously developed diet scores and rapid screeners do not capture many of the key cholesterol-lowering foods that make up the Portfolio Diet. Moreover, as tailoring to a patient’s dietary preferences is considered a key strategy for improving adherence (33, 34), having multiple evidence-based nutrition therapies to choose from is important for patients. To the best of our knowledge the c-PDS is the only score for clinical tracking of the Portfolio Diet. We used the original validated Portfolio Diet Score (PDS), developed by Glenn et al. (35) used in epidemiological settings to inform the development of the c-PDS in the current study. The original PDS is a population-based dietary score ranging from 6 to 30 points for measuring exposure to the Portfolio Diet in prospective cohort studies (35–38). While developing the patient-facing c-PDS, it was decided that the PDS’s sixth category of foods high in saturated fat and cholesterol (which is weighed negatively), would not be included in the score. The omission of the negative category was to ensure the c-PDS was easy for patients to use with concerns raised in consultation with study dietitians that reverse scoring would lead to confusion. Moreso, the positive focus on the five categories is consistent with previous messaging given to participants in the Portfolio Diet intervention trials. Another difference between the scores was the plant sterol category. To ensure the c-PDS was in keeping with advice given in an intervention trial (target of 2 grams/2000 kcal) only supplements or fortified foods were included in the c-PDS such as a fortified plant-sterol margarine and plant-sterol sachets.

### 4.2 Strengths and limitations

In our present study, we assessed the ability of the c-PDS to predict concomitant changes in LDL-C, which has been extensively studied and has been described as a causal for CVD (39). Moreover, LDL-C is the primary target of the Portfolio Diet. Another strength was the use of multiple linear regression analysis which allows for possible confounders to be accounted for and predictions about health outcomes to be made. By undertaking this analysis, we estimated that a reduction in LDL-C of 0.53 mmol/L (13.1%) may be observed with a 12-point increase in the c-PDS. Furthermore, our analysis was performed in the Portfolio Diet’s target population of adults with hyperlipidemia, strengthening our confidence in our estimated benefit. However, it is important to note that this was a secondary analysis, and therefore no direct assessment of benefit to participants can be made. Another limitation of the present study was the use of self-reported dietary records, which may not reflect true dietary intake. Measurement errors in self-reported dietary data can result in systematic bias such as intake-related bias where over-reporting of healthy foods and under-reporting of unhealthy foods may occur due to social pressures (40). This issue of self-reported data remains a major challenge in all nutrition research (41). A separate limitation of this study, when examining the five individual categories of the diet, the High MUFA Oils and Foods category was not associated with LDL-C or HDL-C. However, no change in this category was observed over the 6-month period, so no association with HDL-C could be established. Furthermore, the current study that the sample of participants was only from a single study and was made up of predominately White adults, limiting the generalizability of the findings to other populations. Future research in populations with different demographic characteristics from those in this study, including underrepresented populations, is needed (42).

### 4.3 Practical application of the c-PDS in clinical settings

While nutrition and lifestyle therapy are the major cornerstones of preventative therapy for cardiovascular disease (8–12) effectively providing nutrition therapy in clinical settings is challenging. As supporting material to the Portfolio Diet infographic, currently hosted on the Canadian Cardiovascular Society’s (CCS) website (43), the c-PDS can be used in clinical settings to facilitate self-tracking through the patient-facing score sheet (Supplementary Figure S4). Short screeners and scoring tools, easily used in various settings, can enhance dissemination (18). Additionally, self-tracking or monitoring is essential for effective interventions, as it is a cornerstone of behavior change techniques and is essential for producing sustained behavior change (44) such as dietary self-monitoring food intake for supporting weight loss (45).

Beyond screeners, tools for dietary self-tracking that allow for the combination of self-tracking with other behavior change techniques are essential for effective long-term interventions (44), empowering both patients and healthcare providers to embrace dietary interventions. This led us to mount the c-PDS within the PortfolioDiet.app, a freely available health application for delivering the Portfolio Diet in clinical settings (46). As a transformative digitally-enabled health services tool for the translation of current CCS guidelines (8), the PortfolioDiet.app was designed to help patients manage dyslipidemia and prevent CVD. The c-PDS is used in the PortfolioDiet.app to translate dietary adherence data into tailored feedback for patients, a critical addition to self-monitoring for behavioral interventions (47).

### 4.4 Implications and future directions

Because CVD remains the leading cause of mortality globally (1), there are significant implications of expanding access to the Portfolio Diet. As evidence continues to emphasize how critical the cumulative exposure over one’s lifetime to atherogenic particles is for CVD risk (39, 48, 49), the role for early long-term adoption of a healthy dietary pattern to improve risk is evident. This emphasis is supported by evidence from 3 prospective cohorts found higher adherence to the Portfolio Diet over 30 years was associated with a 14% lower risk of total CVD (50). While a number of evidence-based therapeutic dietary patterns have been developed for CVD, creative solutions to help support people to follow these dietary patterns are needed.

We hope this work serves as an example for other healthy dietary patterns and highlights the potential of user-friendly scores to help translate nutrition. The PortfolioDiet.app uses the c-PDS to provide the user with personalized feedback through the interactive dashboard displays, goal settings, Short Message Service (SMS) texts, and gamification components such as leaderboards. By undertaking this analysis on the c-PDS, we can predict that if patients achieve a 12-point increase in the c-PDS, they can anticipate a clinically meaningful reduction in their LDL-C. Supplementary Figure S9 provides an example of how the 12/25 points target has informed how we frame our messaging and feedback to patients within the PortfolioDiet.app, allowing for positive messaging with a goal of 12/25 points (~50% adherence). Importantly, a relatively similar level of adherence to the Portfolio Diet (~46%) was found in a multi-center trial, which significantly lower LDL-C by ~13% over 6 months (6).

While developing the PortfolioDiet.app, considerations to ensure the best integration of the c-PDS were made. Because the c-PDS uses the EER to allow for personalization of dietary goals, to reflect this characteristic in the PortfolioDiet.app, an EER question was built into the patient-facing side of the app. Using four patient-entered variables, the app automatically calculates EER and allocates users to their respective calorie group with corresponding c-PDS targets. Beyond this, the app allows tracking of the c-PDS as well as other health measures (LDL-C, fasting blood glucose, and blood pressure). By providing patients with a tool to track their behaviors and health outcomes, this tool allows them to draw a relationship between the two, improving self-efficacy. Findings from a quality improvement and usability study found the PortfolioDiet.app was considered acceptable by users and that the c-PDS used in the app did not cause confusion (46). Based on these previous findings and the current study’s findings, we anticipate the c-PDS as part of the PortfolioDiet.app aid in the dissemination of the Portfolio Diet. Future research in a randomized controlled trial will assess if the c-PDS, as part of the PortfolioDiet.app, is effective in promoting behavior change and subsequent health-related outcomes, such as lipid targets.

## 5 Conclusion

By facilitating (self-) tracking of the Portfolio Diet by participants and clinicians, we anticipate that the c-PDS will aid in the uptake of the Portfolio Diet under the wide range of circumstances that constitute clinical practice. Undertaking this analysis on the c-PDS, allowed us to predict that if patients achieve a 12-point increase in the c-PDS, they can anticipate a clinically meaningful reduction in LDL-C of 0.53 mmol/L (13.1%). This 12/25 points target has been incorporated into the messaging and goals to patients within the PortfolioDiet.app, a clinical health application for delivering the Portfolio Diet. Future research will assess if the c-PDS when used as part of the PortfolioDiet.app in a randomized controlled trial is effective in promoting behaviour change and subsequent health-related outcomes, such as desired lipid targets in clinical practice.

## Data availability statement

The data analyzed in this study is subject to the following licenses/restrictions: Publicly available data are contained within the article. Requests to access these datasets should be directed to John.sievenpiper@utoronto.ca.

## Ethics statement

The studies involving humans were approved by Research Ethics Committee of St. Michael’s Hospital (REB#04–056). The studies were conducted in accordance with the local legislation and institutional requirements. The participants provided their written informed consent to participate in this study.

## Author contributions

MK: Conceptualization, Formal analysis, Investigation, Methodology, Writing – original draft, Writing – review & editing. AG: Conceptualization, Formal Analysis, Methodology, Writing – original draft, Writing – review & editing. LC: Conceptualization, Methodology, Writing – original draft, Writing – review & editing. GM: Writing – original draft, Writing – review & editing. RJ: Conceptualization, Methodology, Writing – original draft, Writing – review & editing. VM: Conceptualization, Methodology, Writing – original draft, Writing – review & editing. CM: Methodology, Writing – original draft, Writing – review & editing. CK: Data curation, Methodology, Writing – original draft, Writing – review & editing. DJ: Conceptualization, Data curation, Methodology, Writing – original draft, Writing – review & editing. JS: Conceptualization, Funding acquisition, Methodology, Supervision, Writing – original draft, Writing – review & editing.
