# Supplementary material for: Simulation model to assess the validity of the clinical portfolio diet score used in the PortfolioDiet.app for dietary self-tracking: a secondary analysis of a randomized controlled trial in hyperlipidemic adults
Source: Front Nutr. 2024 Aug 7;11:1398450. doi: 10.3389/fnut.2024.1398450 (PMC11335621; doi:10.3389/fnut.2024.1398450)
Supplement: Supplementary file 1 [file Data_Sheet_1.pdf]

Supplementary Material

Simulation model to assess the validity of the clinical Portfolio Diet Score used in the PortfolioDiet.app for dietary self-tracking: a secondary analysis of a randomized controlled trial in hyperlipidemic adults

Supplemental material contents:

Figure. S1. Flowchart for validation study sample selection 2

Figure. S2. 2,000kcal version of the original food-based screener 3

Figure. S3. Decision tree for scoring foods from 7DDRs to the clinical Portfolio Diet Score 4

Table S1: Portfolio Diet components with the c-PDS category targets in reasonable household measures recommended for 1,200, 1,600, and 2,000 kcal diets. 5

Figure S4: Example of the c-PDS patient-facing score sheet for a 2000kcal diet 8

Table S2. Characteristics of the participants included in the validation analyses. 11

Table S3: Bland-Altman analysis by c-PDS and reference method for categories of the Portfolio Diet 13

Table S4: Predictive validity shown by food components of the c-PDS using % change (week 0 to 24) of LDL-C as a biomarker of adherence 14

Figure S4-8: Scatterplots (a) and Bland Altman plots (b) for each of the Portfolio Diet categories 15

Figure S9: PortfolioDiet.app dashboard display example of the 12/25 messaging using the cPDS. 18

Figure. S1. Flowchart for validation study sample selection

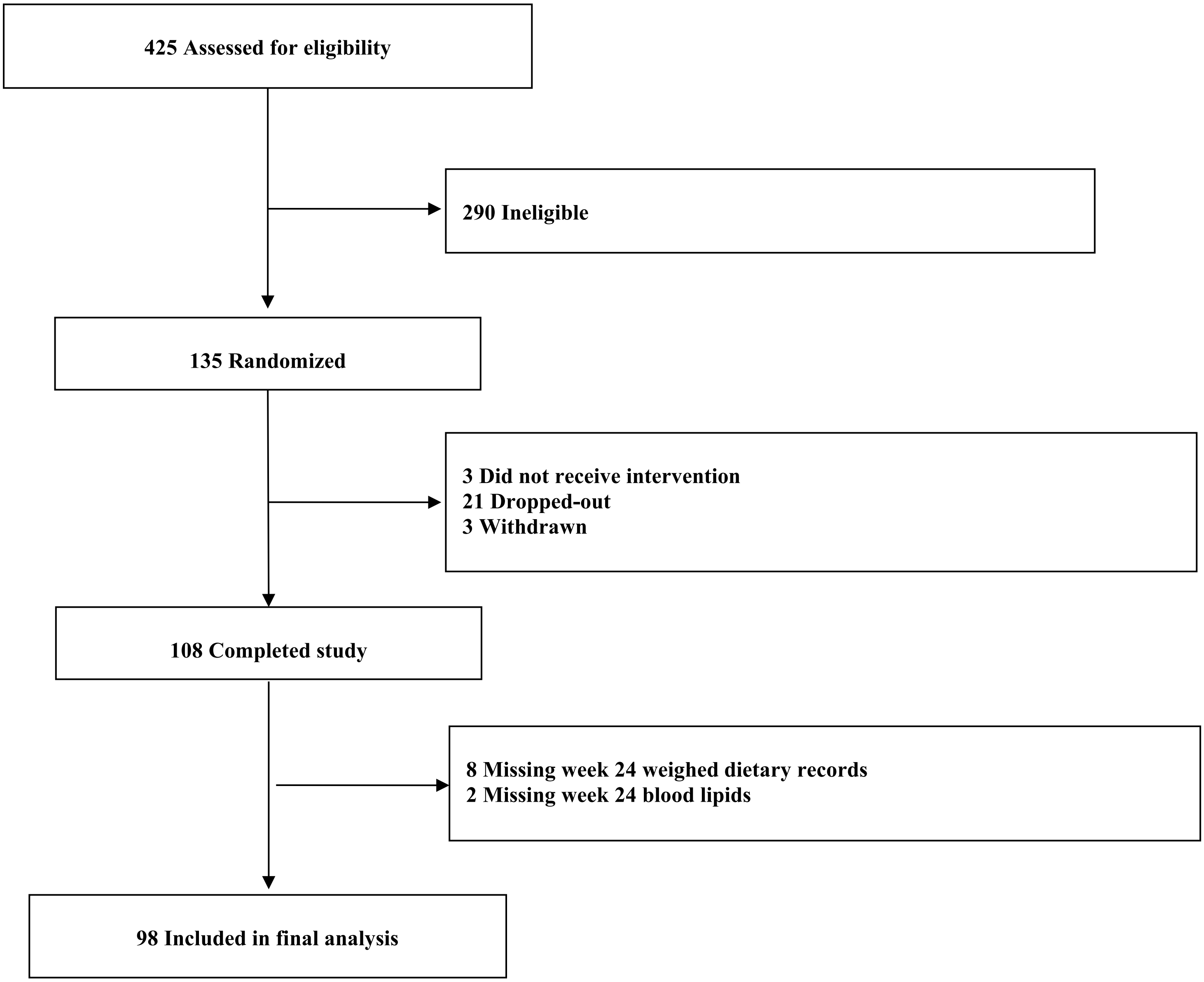

A total of 135 participants were randomized at the Toronto site. Of the 108 participants that completed the study, 8 had missing diet records and 2 had missing blood lipid data. Therefore a total of 98 participants were included in the final validation analysis.

Figure. S2. Original food-based screener used in the Portfolio Diet trials for a 2,000kcal diet

Daily Checklist for Required Enhanced Dietary Portfolio Components **2000 Kcal**

Required components		Required servings per day	Examples of Single Servings	Day 1	Day 2	Day 3	Day 4	Day 5	Day 6	Day 7	My total weekly servings
**Viscous Fibre**		**7**	**Steel cut oats** (dry)—½ cup								
			**Oat bran** (dry)—½ cup								
			**Oat bran bread**—1 slice								
			**All bran Buds w psyllium**—□ cup								
			Barley (dry)—¼ cup								
			Psyllium husk—2 tsp								
			Okra (frozen)—½ cup (□ svg)								
			Eggplant (raw)—2 cups (□ svg)								
**Plant protein**	**Soy protein**	**7**	**PC-BM vegetarian Chicken breast**—½ piece								
			**Soy beverage (fortified)** —1cup								
			Tofu (extra firm, low fat)—¼ cup								
			Soy deli slices—4 slices								
			Soy burgers—1 whole								
			Soy hot dogs—1 whole								
	**Pulses**	**2**	**Beans (cooked/canned)**—½ cup								
			**Chickpeas (cooked/canned)**—½ cup								
			**Lentils (cooked/canned)**—½ cup								
			**Peas (cooked/canned)**—½ cup								
**Plant sterols**		**5**	**Pro-activ spread**—1 tsp								
**Nuts**		**1.5**	**Almonds**—24 nuts (1 handful)								
			**Walnuts**—14 halves								
			**Peanuts**—28 nuts								
**Oils & high MUFA foods** (mono unsaturated fatty acid)		**4**	**Olive Oil (cold pressed)**—1 tsp								
			**Canola Oil (cold pressed)**—1 tsp								
			**Soy bean Oil (cold pressed)**1 tsp								
			Other high MUFA Oils—1tsp:								
			Sunflower, Safflower, Peanut								
			Avocado—28g; ~ 2Tbsp pureed								
			**Nuts** ~ 7g (½Tbsp. nut butter)								
**Low GI Foods**	**Pasta**	**1**	**Pasta** (al dente)–½ cup								
	**Temperate fruits**	**5**	Apple—1 medium)								
			Orange—1 (medium)								
			Berries: Blue/black/ strawberries etc.— ½ cup								
**My total daily servings:**											

Figure. S3. Simulation model decision tree for scoring the clinical Portfolio Diet Score from 7-day diet records

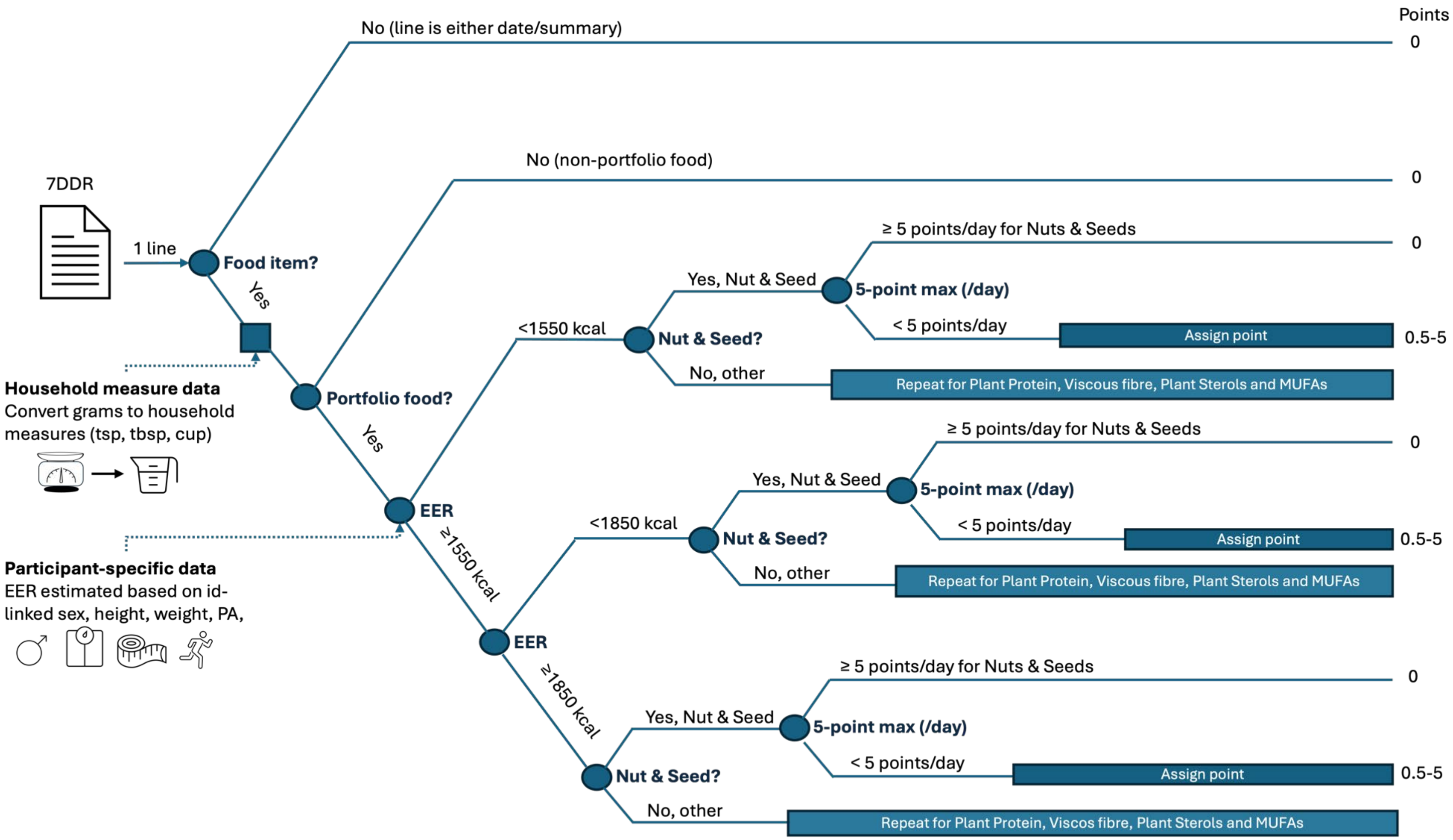

Abbreviations: 7DDR, 7-day diet record; EER, estimated energy requirement. Decision tree illustration for a simulation model executed utilizing the weighed 7DDRs and participant-specific data. From the 7DDRs, the model identified relevant food items from non-food items and then possible portfolio foods from non-portfolio foods. The recorded weighed food intake from the 7DDRs was converted to household measurements. Subsequently, individual participants were allocated to respective calorie groups (1200, 1600 or 2000kcal) based on their EER, corresponding to adjustments of their respective c-PDS targets. Shown here is the decision tree for Nuts & Seeds. Portfolio foods were classified into a total of five distinct categories: Nuts & Seeds, Plant Protein, Viscous Fibre, Plant Sterols, and High MUFA Oils & Foods.

Table S1: Portfolio Diet categories with the c-PDS targets in reasonable household measures for 1,200, 1,600, and 2,000 kcal diets.

Portfolio Diet Categories	Examples of household measures for 1 c-PDS point		
	**1,200 kcal**	**1,600 kcal**	**2,000 kcal**
**Nuts & Seeds, amount/point**	Almonds - 5 nuts	Almonds - 6 nuts	Almonds - 7 nuts
	Walnuts - 3 halves	Walnuts - 3 halves	Walnuts - 4 halves
	Pistachio (shell-free) - 10 nuts	Pistachio (shell-free) - 12 nuts	Pistachio (shell-free) - 15 nuts
	Peanuts (shell-free) - 6 nuts	Peanuts (shell-free) - 7 nuts	Peanuts (shell-free) - 8 nuts
	Other nuts - 5 nuts	Other nuts - 6 nuts	Other nuts - 7 nuts
	Nut butters, all natural - 1 tsp	Nut butters, all natural - 1.5 tsp	Nut or seed butters, all natural - 2 tsp
	Ground flax seeds - 2 tsp	Ground flax seeds - 1 tbsp	Ground flax seeds - 1 tbsp
	Seeds, e.g. pumpkin, sunflower - 2 tsp	Seeds, e.g. pumpkin, sunflower - 1 tbsp	Seeds, e.g. pumpkin, sunflower, chia - 1 tbsp
**Plant Protein, amount/point**	Soy/Pea beverage (fortified) - 3/4 cup	Soy/Pea beverage (fortified) - 1 cup	Soy/Pea beverage (fortified) - 1.25 cups
	Tofu (extra firm or firm) - 1/4 cup	Tofu (extra firm or firm) - 1/3 cup	Tofu (extra firm or firm) - 1/2 cup
	Tofu (silken, soft) - 1/3 cup	Tofu (silken, soft) - 1/2 cup	Tofu (silken, soft) - 2/3 cup
	Tempeh - 1/8 package (29g)	Tempeh - 1/6 package (33g)	Tempeh - 1/4 package (50g)
	Edamame (shelled) - 1/4 cup	Edamame (shelled) - 1/3 cup	Edamame (shelled) - 1/2 cup
	Chickpeas (cooked/canned) - 1/3 cup	Chickpeas (cooked/canned) - 1/2 cup	Chickpeas (cooked/canned) - 1/2 cup
	Beans (cooked/canned) - 1/3 cup	Beans (cooked/canned) - 1/2 cup	Beans (cooked/canned) - 1/2 cup
	Lentils (cooked/canned) - 1/3 cup	Lentils (cooked/canned) - 1/2 cup	Lentils (cooked/canned) - 1/2 cup

	Peas, green - 2/3 cup	Peas, green - 3/4 cup	Peas, green - 1 cup
	Veggie burger - 1/2 patty*	Veggie burger - 1 patty*	Veggie burger - 1 patty*
	Veggie sausage patties - 1 patty*	Veggie sausage patties - 1 1/2 patties*	Veggie sausage patties - 2 patties*
	Veggie meat grounds - 1/5 cup*	Veggie meat grounds - 1/4 cup*	Veggie meat grounds - 1/3 cup*
	Chick'n tenders - 2 tenders*	Chick'n tenders - 2 tenders*	Veggie Chick'n tenders - 3 tenders*
	Soy/pea protein powders** - 1 tbsp	Soy/pea protein powders** - 1.5 tbsp	Soy/pea protein powders** - 1.5 tbsp
**Viscous Fibre, amount/point**	Psyllium husk - 1 tsp	Psyllium husk - 1 tsp	100% Psyllium husk powder - 1 tsp
	100% Psyllium husk - 1 tbsp	100% Psyllium husk - 1 tbsp	100% Psyllium husk - 1 tbsp
	Psyllium powder product (e.g. Metamucil) - 2tsp	Psyllium powder product (e.g. Metamucil) - 2tsp	Psyllium powder product (e.g. Metamucil) - 2tsp
	PGX supplement - 2g (2-3 capsules)	PGX supplement - 2g (2-3 capsules)	PGX supplement - 2g (2-3 capsules)
	Oat bran (dry) - 1/2 cup	Oat bran (dry) - 1/2 cup	Oat bran (dry) - 2/3 cup
	All-bran Buds with Psyllium - 1/4 cup	All-bran Buds with Psyllium - 1/3 cup	All-bran Buds with Psyllium - 1/2 cup
	Steel cut oats/oatmeal (dry) – 1/2 cup	Steel cut oats/oatmeal (dry) – 2/3 cup	Steel cut oats/oatmeal (dry) – 3/4 cup
	Oat bran bread§ - 3/4 slice	Oat bran bread§ - 1 slice	Oat bran bread§ - 1 slice
	Barley (cooked) - 1 cups	Barley (cooked) - 1.5 cups	Barley (cooked) - 2 cups
	Okra (cooked) - 2 cups	Okra (cooked) - 2.5 cups	Okra (cooked) - 3 cups
	Eggplant (cooked) - 3 cups	Eggplant (cooked) - 4 cups	Eggplant (cooked) - 4 cups
	Persimmons - 1 large	Persimmons - 1.5 large	Persimmons - 2 large
	Oranges - 2 large	Oranges - 2 large	Oranges - 2 large
	Apples - 2 large	Apples - 2 large	Apples - 2 large

	Berries - 2 cups	Berries - 2 cups	Berries - 2 cups
**Plant Sterols, amount/point**	Plant sterol margarine - 1 tsp	Plant sterol margarine - 1 tsp	Plant sterol margarine - 1 tsp
	Packet - 1/5th packet	Packet - 1/5th packet	Packet - 1/5th packet
	Plant sterol powder - 1/5 tsp	Plant sterol powder - 1/5 tsp	Plant sterol powder - 1/5 tsp
	Supplements - 1 capsule¥	Supplements - 1 capsule¥	Supplements - 1 capsule¥
**High MUFA Oils & Foods, amount/point**	Extra virgin Olive Oil - 1 tsp	Extra virgin Olive Oil - 1.5 tsp	Extra virgin Olive Oil - 2 tsp
	Cold press Canola Oil - 1 tsp	Cold press Canola Oil - 1.5 tsp	Cold press Canola Oil - 2 tsp
	Cold press Soy Bean Oil - 1 tsp	Cold press Soy Bean Oil - 1.5 tsp	Cold press Soy Bean Oil - 2 tsp
	High Oleic Oils - 1 tsp: (E.g. High Oleic Sunflower, Safflower or Soy)	High Oleic Oils - 1.5 tsp (E.g. High Oleic Sunflower, Safflower or Soy)	High Oleic Oils - 2 tsp (E.g. High Oleic Sunflower, Safflower or Soy)
	Canola Oil - 1 tsp	Canola Oil - 1.5 tsp	Canola Oil - 2 tsp
	Soybean Oil - 1 tsp	Soybean Oil - 1.5 tsp	Soybean Oil - 2 tsp
	Avocado - 1/8 fruit	Avocado - 1/6 fruit	Avocado - 1/4 fruit

* For plant-based meats, they must come from soy or legume based proteins. Avoid products with mostly wheat or gluten proteins, and also avoid jackfruit-based plant proteins.
**For protein powder, it must come from soy or legume based proteins. Avoid rice and other grain-based proteins.
§ See Oatbran Bread Recipe, enriched in psyllium fibre.
¥ For Supplements, typically 5 capsules = 2g plant sterols which is the total recommended amount per day.

The table shows the five Portfolio Diet components: Nuts & Seeds, Plant Protein, Viscous Fibre, Plant Sterols, and High MUFA Oils & Foods, and their respective examples of foods for 1 point by each EER groups. Target amounts for 1-point of the c-PDS were food-based and provided in reasonable household measurements. For each category of the c-PDS, points were rounded to the nearest 0.5, i.e., ½ cup chickpeas has ~ 10 grams protein = 1 point for plant protein for those with an EER of 2000kcal. A maximum of 5-points was set for each category (total score ranged from 0 to 25-points).

Figure S4: Example of the developed patient-facing score sheet for a 2000kcal diet

Clinical-based Portfolio Diet Score (c-PDS) Checklist — 2,000 Kcal

Daily Checklist for Measuring Your Adherence to the Portfolio Diet.

There are 5 key categories, and each category is worth 5 points. The highest score possible is 25/25 points.

Tally the foods you have eaten today that are on this list below to get your Portfolio Diet Score.

Portfolio Diet Categories	Food	Criteria for **1 point**	Tally your points e.g. 𝍸	Total points for each category
NUTS & SEEDS 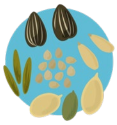	Almonds	9 nuts (9g)		
	Walnuts	5 halves (9g)		
	Pistachios	15 nuts (9g)		
	Peanuts (shell-free)	9 nuts (9g)		
	Other nuts	9 nuts (9g)		
	Nut butters, all natural	2 tsp		
	Seeds, e.g. pumpkin, sunflower	1 tbsp		
	**Max total points to aim for:**		☐ ☐ ☐ ☐ ☐	= ___ / 5
PLANT PROTEIN	Soy or Pea beverage (fortified)	1.25 cups		
	Tofu (extra firm or firm)	1/2 cup (100g)		
	Tofu (silken, soft)	3/4 cup (170g)		

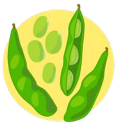	Tempeh	1/4 package (50g)		
	Edamame (shelled)	1/2 cup (95g)		
	Veggie burger	1 patty (75g)		
	Veggie sausage patties	2 patties (57g)		
	Veggie meat grounds	1/3 cup (55g)		
	Veggie chick'n tenders	3 tenders (75g)		
	Beans	1/2 cup		
	Peas, green	1 cup		
	Chickpeas	1/2 cup		
	Lentils	1/2 cup		
	Soy or Pea protein powders	1.5 tbsp (12g)		
	**Max total points to aim for:**		☐ ☐ ☐ ☐ ☐	= ___ / 5
VISCOUS FIBRE 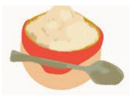	Psyllium husk	5g (1 tbsp)		
	100% Psyllium husk powder	5g (1 tsp)		
	Psyllium powder product (e.g. Metamucil)	10 g (2 tsp)		
	PGX supplement	2 g (2-3 capsules)		
	Oat bran (dry)	2/3 cup (63g)		
	All-bran Buds with psyllium	1/2 cup (45g)		
	Steel cut oats/oatmeal (dry)	3/4 cup (119g)		

	Oat bran bread[§]	1 slice (65g)		
	Barley (cooked)	2 cups (314g)		
	Okra (cooked)	3 cups (480g)		
	Eggplant (cooked)	4 cups (328g)		
	Oranges	2 large		
	Apples	2 large		
	Berries	2 cups		
	Persimmons	2 large		
	**Max total points to aim for:**		□ □ □ □ □	= ___ / 5
PLANT STEROLS 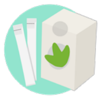	Plant sterol margarine	1 tsp		
	Plant sterol packet<br><br>(1 packet = 5 points)	1/5th packet		
	Plant sterol supplement	~1 capsule		
	**Max total points to aim for:**		□ □ □ □ □	= ___ / 5
OILS (MUFAS) 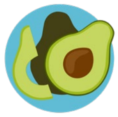	Extra virgin olive oil	2 tsp		
	Cold press canola oil	2 tsp		
	Cold press soybean oil	2 tsp		
	High oleic oils:<br>High oleic sunflower, safflower or soybean	2 tsp		
	Canola oil	2 tsp		
	Soybean oil	2 tsp		

	Avocado	1/4 fruit (50g)		
	Max total points to aim for:		☐ ☐ ☐ ☐ ☐	= ___ / 5
	**TOTAL** DAILY PORTFOLIO DIET POINTS (Portfolio Diet Score)			___ / 25

Your target is a Portfolio Diet Score of **12/25**. By reaching this target regularly you will lower your LDL-cholesterol by 10-15%. This lowering is within the range you can expect with some cholesterol-lowering medications.

Table S2. Characteristics of the participants included in the validation analyses

**Characteristics, mean (SD) / No. (%)**		n = 98
Age, years		56.36 ± 9.05
Sex, No. (%)		
	Female	59 (60)
	Male	39 (40)
Ethnicity, No. (%)		
	White	64 (65)
	Asian	18 (18)
	Black	9 (9)
	Hispanic	1 (1)
	Other[a]	6 (6)
Body weight, kg		74.41 ± 14.00
BMI, kg/m^2		27.1 ± 4.2
BP, mmHg		
	Systolic BP	116.32 ± 11.03
	Diastolic BP	71.23 ± 7.89
Lipids, mmol/L		
	LDL-C	4.33 ± 0.80
	Non-HDL-C	4.98 ± 0.89
	Total cholesterol	6.30 ± 0.93
	HDL-C	1.32 ± 0.34
	Triglycerides	1.49 ± 0.90
ApoB, g/L		1.20 ± 0.19
hsCRP, mg/L		1.87 ± 4.73
Medication use, No. (%)		
	Lipid-lowering medication[b]	14 (14)

Anti-hypertensive medication	15 (15)
Hormone therapy medication	1 (1)
Thyroxine	8 (8)
Daily calories, kcals	1973.80 ± 500.41
Adherence to the Portfolio Diet, % (±SD)[c]	18.30 ± 10.01
c-PDS, points	3.11 ± 2.64

Data are shown as mean (SD) or No. (%). Abbreviations: ApoB, Apolipoprotein B; BMI, body mass index; BP, blood pressure; c-PDS, clinical Portfolio Diet Score; hsCRP, highly sensitive c-reactive protein; HDL-C, high-density lipoprotein cholesterol; LDL-C, low-density lipoprotein cholesterol.

Means (±SD), unless otherwise noted

[a] Includes those of mixed or unknown.
[b] Participants were required to discontinue lipid-lowering medication 2 weeks prior to randomization.
[c] Method used traditionally to assessed adherence to the Portfolio Diet in trials from 7DDRs.

To convert total cholesterol, HDL, non-HDL-C, and LDL-C from mmol/L to mg/dL multiply by 38.67.
To convert triglycerides from mmol/L to mg/dL multiply by 88.57.
To convert CRP mg/L to mg/dL divide by 10.

Table S3: Bland-Altman analysis by c-PDS and reference method by categories of the Portfolio Diet

Portfolio Diet Component	Mean difference (95% CI)*	Limits of agreement**
**Nuts & Seeds**	-5.80 (-8.35, -3.25)	-31.26 to 19.67
**Plant Protein**	-3.00 (-6.0, -0.02)	-32.78 to 26.77
**Viscous Fibre**	5.00 (1.74, 8.27)	-27.52 to 37.53
**Plant Sterols**	-3.76 (-5.61, -1.90)	-22.27 to 14.76
**High MUFA Oils & Foods**	-14.63 (-19.08, -10.19)	-59.00 to 29.74
**Total c-PDS**	-4.44 (-6.31, -2.57)	-23.10 to 14.22

*Data is shown by percentage
**95 % limits of agreement

Table S4: Predictive validity shown by Portfolio Diet categories using cPDS and % change of LDL-C (from week 0 to 24) as a biomarker of adherence.

		Unadjusted		Adjusted**	
**Portfolio Diet categories**	**Average change (95% CI)**	**β Coefficients* (95%CI)**	***p*-Value**	**β Coefficients* (95%CI)**	***p*-Value**
**Nuts & Seeds**	0.45 (0.05, 0.86)	-3.12 (-4.62, -1.62)	<0.001	-2.21 (-3.60, -0.82)	0.002
**Plant Protein**	0.94 (0.65, 1.22)	-3.51 (-5.69, -1.33)	0.002	-3.80 (-5.75, -1.85)	<0.001
**Viscous Fibre**	1.06 (0.71, 1.41)	-4.14 (-5.82, -2.46)	<0.001	-3.41 (-4.97, -1.86)	<0.001
**Plant Sterols**	1.43 (1.10, 1.76)	-4.00 (-5.83, -2.16)	<0.001	-3.21 (-4.89, -1.53)	<0.001
**High MUFA Oils & Foods**	0.03 (-0.17, 0.22)	-0.10 (-3.47, 3.27)	0.953	-0.66 (-3.65, 2.33)	0.664
**Total c-PDS**	3.91 (2.74, 5.07)	-1.28 (-1.78, -0.78)	<0.001	-1.09 (-1.55, -0.63)	<0.001

*Data is shown for change in LDL-C expressed as percentage for a 1-point change in the c-PDS by Portfolio Diet categories.
**Model adjusted for age, sex, BMI, ethnicity, and baseline LDL-C

Figure S4-8: Scatterplots (a) and Bland Altman plots (b) for each of the Portfolio Diet categories

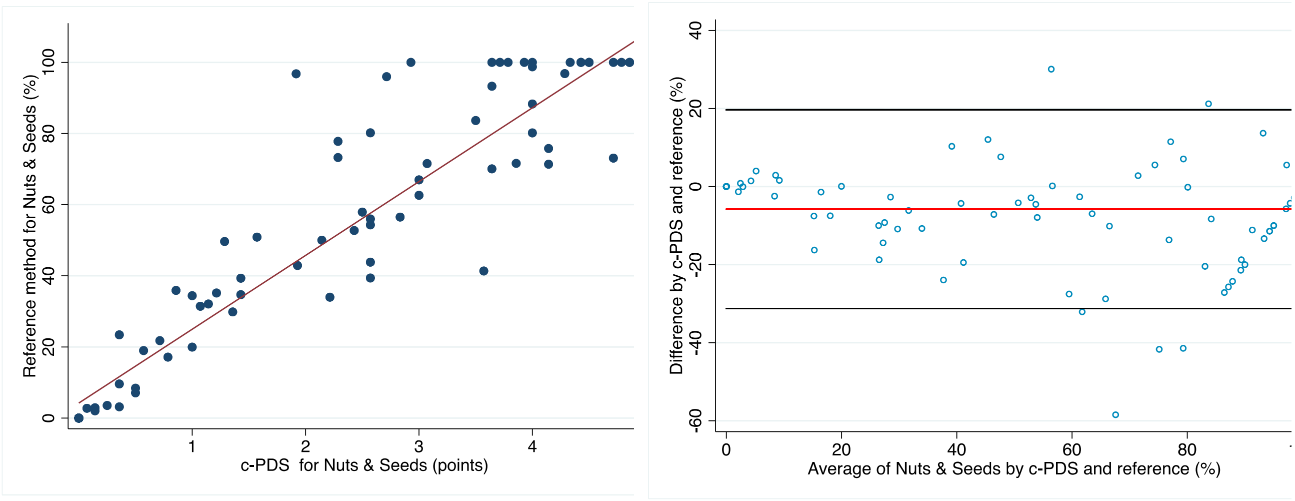

(**a**) (**b**)

Figure S4. (a) A scatter plot of the dietary adherence to the Nuts & Seeds category measured by the reference method, shown as percentage, and the c-PDS, shown as points (range, 0 to 25-points). **(b)** Bland-Altman plot of agreement between the c-PDS with the reference method on % adherence to Nuts & Seeds assessed by weighed 7-day diet records (week 24). The red line is the mean difference and black lines represent upper and lower 95 % limits of agreement. Abbreviations: c-PDS, clinical-Portfolio Diet Score.

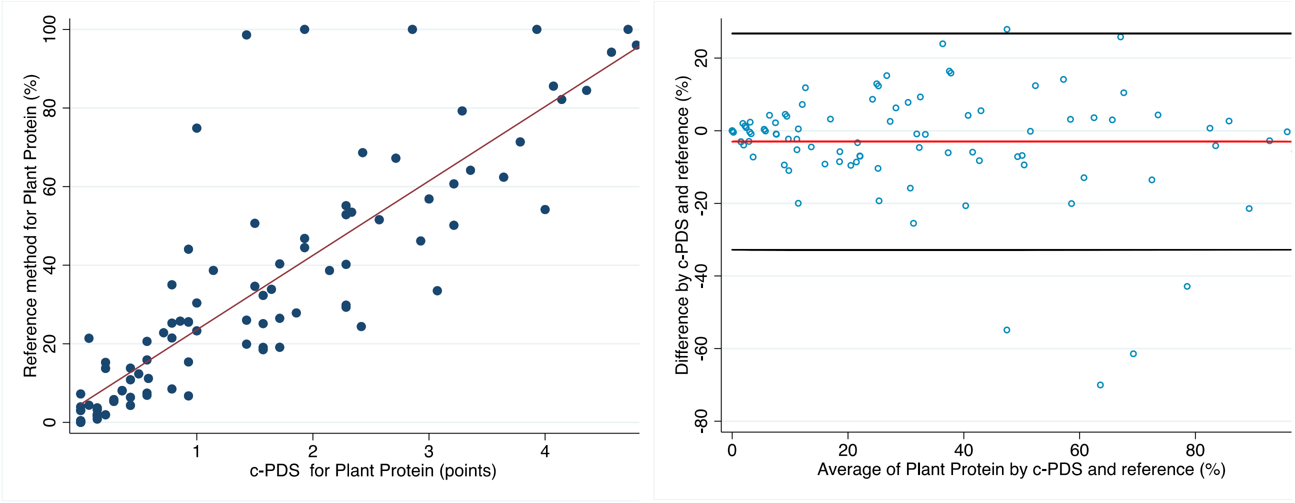

(**a**) (**b**)

Figure S5. (a) A scatter plot of the dietary adherence to the Plant Protein category measured by the gold-standard reference method, shown as percentage, and the c-PDS, shown as points (range, 0 to 25-points). **(b)** Bland-Altman plot of agreement

between the c-PDS with the reference method on % adherence to Plant Protein assessed by weighed 7-day diet records (week 24). The red line is the mean difference and black lines represent upper and lower 95 % limits of agreement. Abbreviations: c-PDS, clinical-Portfolio Diet Score.

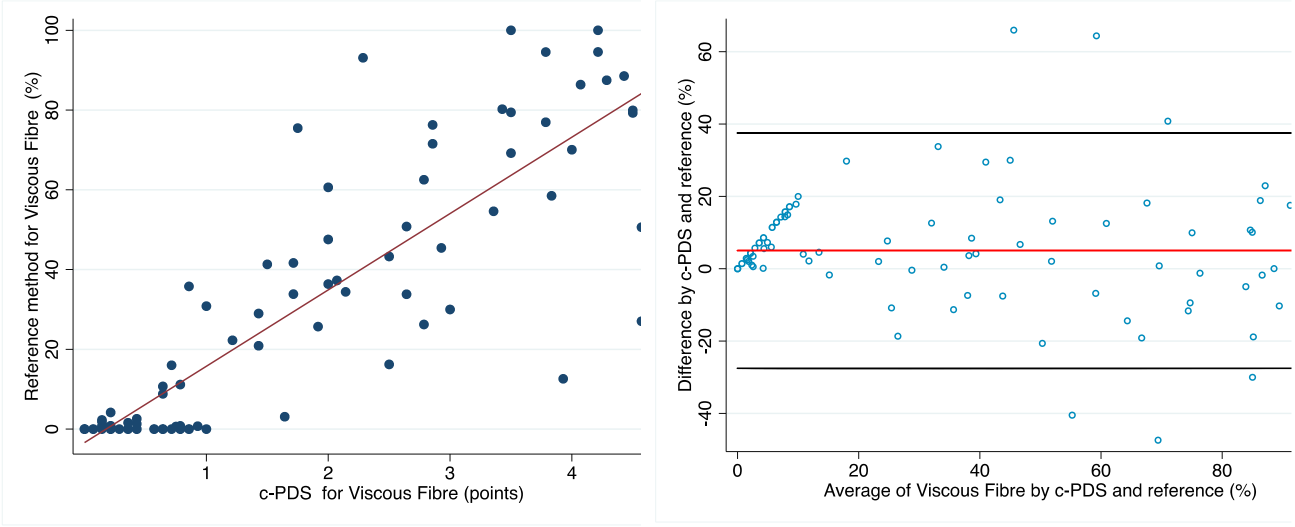

(a) (b)

Figure S6. (a) A scatter plot of the dietary adherence to the Viscous Fibre category measured by the gold-standard reference method, shown as percentage, and the c-PDS, shown as points (range, 0 to 25-points). **(b)** Bland-Altman plot of agreement between the c-PDS with the reference method on % adherence to Viscous Fibre assessed by weighed 7-day diet records (week 24). The red line is the mean difference and black lines represent upper and lower 95 % limits of agreement. Abbreviations: c-PDS, clinical-Portfolio Diet Score.

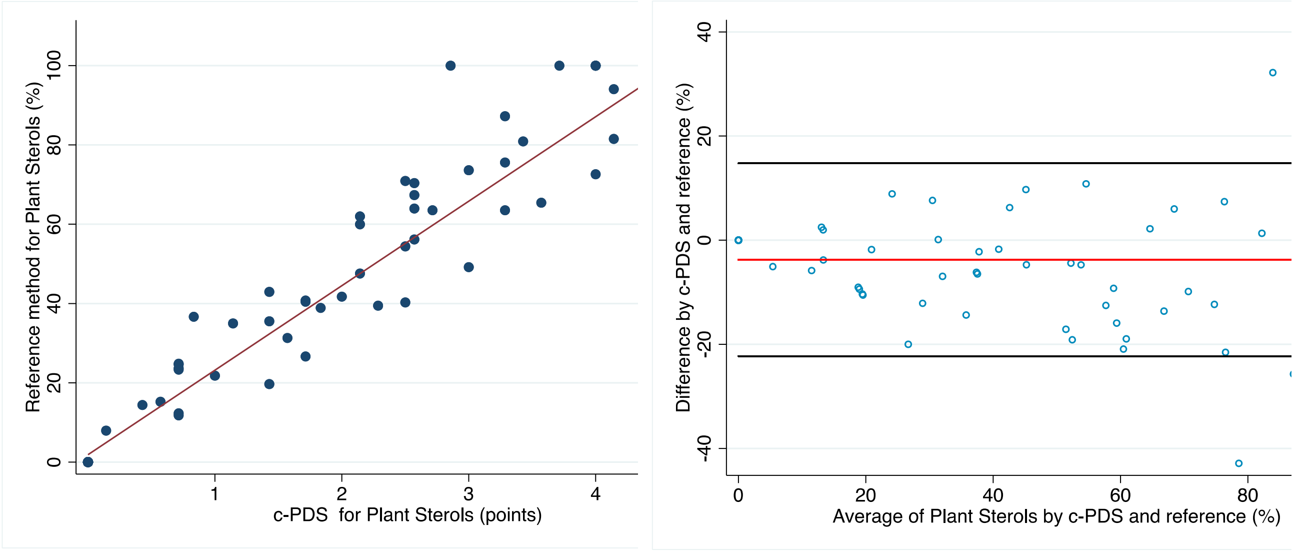

(a) (b)

Figure S7. (a) A scatter plot of the dietary adherence to the Plant Sterols category measured by the gold-standard reference method, shown as percentage, and the c-PDS, shown as points (range, 0 to 25-points). **(b)** Bland-Altman plot of agreement between the c-PDS with the reference method on % adherence to Plant Sterols assessed by weighed 7-day diet records (week 24). The red line is the mean difference and black lines represent upper and lower 95 % limits of agreement. Abbreviations: c-PDS, clinical-Portfolio Diet Score.

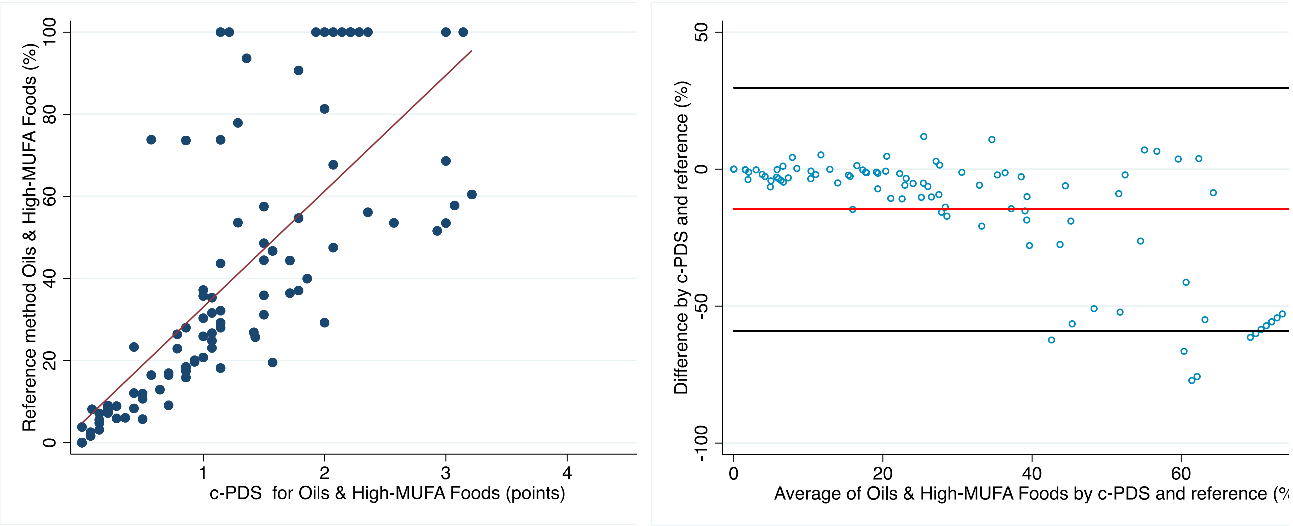

(**a**) (**b**)

Figure S8. (a) A scatter plot of the dietary adherence to the High MUFA Oils & Foods category measured by the gold-standard reference method, shown as percentage, and the c-PDS, shown as points (range, 0 to 25-points). **(b)** Bland-Altman plot of agreement between the c-PDS with the reference method on % adherence to High MUFA Oils & Foods assessed by weighed 7-day diet records (week 24). The red line is the mean difference and black lines represent upper and lower 95 % limits of agreement. Abbreviations: c-PDS, clinical-Portfolio Diet Score.

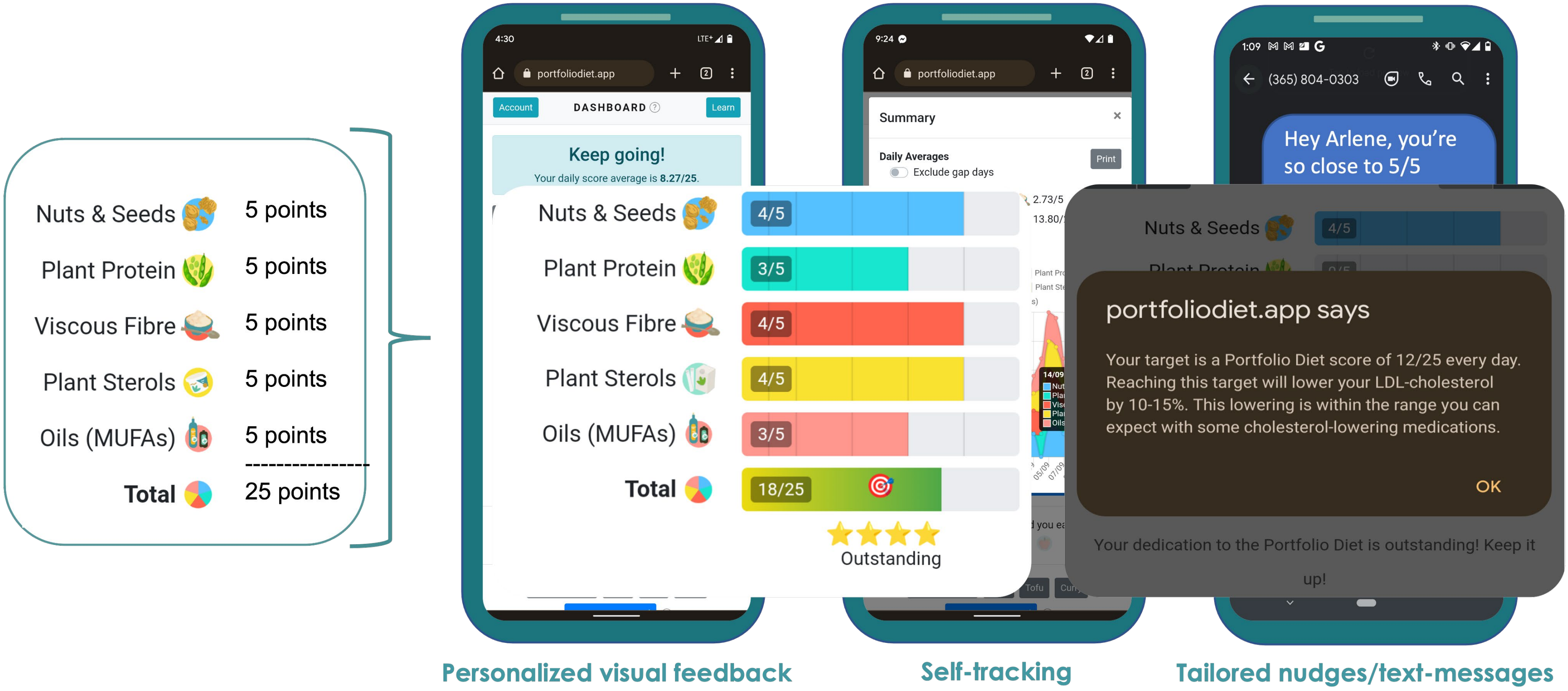

Figure S9: PortfolioDiet.app dashboard display example of the 12/25 messaging using the cPDS.

Example of how the clinical Portfolio Diet Score (cPDS) is used to provide messaging to patients. As indicated on the bar graph using a target symbol in the middle of the total 25-point bar. Once patients reach 12/25 the messaging reflects the goal being achieved, any further points are met with additional praise. If patients click on the target, a message will pop up indicating that by consistently reaching this target they can anticipate a lowering of LDL-cholesterol by 10-15% and that this lowering is within the range you can expect with some cholesterol-lowering medications.
